# The evolution of sex differences in disease

**DOI:** 10.1186/s13293-015-0023-0

**Published:** 2015-03-13

**Authors:** Edward H Morrow

**Affiliations:** Evolution, Behaviour and Environment Group, School of Life Sciences, University of Sussex, John Maynard Smith Building, Falmer, Brighton, BN1 9QG UK

**Keywords:** Sexual dimorphism, Sex-specific genetic architecture, Sex-chromosomes, Evolutionary medicine, Darwinian medicine, Sexual selection, Natural selection, Personalized medicine, Gender medicine

## Abstract

It is now becoming widely recognized that there are important sex differences in disease. These include rates of disease incidence, symptoms and age of onset. These differences between the sexes can be seen as a subset of the more general phenomenon of sexual dimorphism of quantitative phenotypes. From a genetic point of view, this is paradoxical, since the vast majority of genetic material is shared between the sexes. How can males and females differ in so many ways and yet have a common genetic code? Traditionally, the modifying action of hormones has been offered as a solution to this paradox, but experiments disentangling the effects of hormones and sex-chromosomes have shown that this cannot be the sole explanation. In this review, I outline current ideas about the evolutionary origins of sex differences in phenotypes, with a particular focus on how sex differences in disease can arise. I also discuss how sex differences in themselves can generate new risk factors for disease, in effect becoming a new environmental factor, as well as briefly reviewing more general evidence for sexually antagonistic selection and genetic variation within humans. Taking an evolutionary view on sex differences in disease provides an opportunity for greater understanding of mechanisms of disease and as such provides a clear motivation for clinicians to explore how therapies may be tailored to the individual in a sex-dependent way.

## Review

### Introduction

In the inaugural article of *Biology of Sex Differences* [1], Arnold outlined the main motivations for why the existence of separate sexes is an important factor to consider when investigating human disease, the key point is that sex differences in human physiology exist and that they matter, both in terms of determining disease phenotypes and also for shaping more effective therapies. Biomedical scientists have traditionally ignored the importance of phenotypic differences between males and females [1], although this is changing [2]. The NIH, for example, recently announced it will be developing its policies to pay attention to sex differences in preclinical research [3], which is likely to result in considerable benefits should prospective gender-specific therapies be implemented [4].

The effect of sex on disease can be manifest in many ways, including the presentation of the disease and its associated symptoms, the prevalence or age of onset. A review by Ober et al. [5] emphasized that although some sex differences are due to classical differences in circulating hormones, there is increasing evidence that genetic factors make an important contribution. This is illustrated for example by data from the ‘four core genotypes’ model, where hormonal and sex chromosome contributions to phenotypic differences between the sexes can be disentangled from one another [6].

Identifying specific cases of how males and females differ biologically is important therapeutically, but in order for biologists and physicians to gain a full understanding, it is necessary to appreciate how these differences came about [1,7]. Evolutionary theory can provide useful insights into the origins of sex differences, either as adaptations in their own right in the case of physiological differences or in explaining why pathogenic phenotypes persist in a population [7,8].

From an evolutionary standpoint, sex differences in disease can be seen as a subset of the more general phenomenon of sexual dimorphism of quantitative traits, including important life-history traits such as ageing and longevity [9]. It is the ultimate causes and consequences of sexual dimorphism that I explore in this review, with the emphasis on human physiological and disease phenotypes. The aim is to provide a researcher working at the front line of human physiology and disease with a clearer understanding of how sex differences evolve and to explore some of the ways in which sex differences can create novel selective pressures, generating further evolutionary change.

#### The evolution of sexual dimorphism

Evolutionary biologists have long wondered why males and females are different. Darwin’s ‘other book’ *The Descent of Man* catalogs the multitude of ways in which males and females of many different species differ from one another in terms of their morphology and behaviour [10], although not explicitly their physiology. In that volume, Darwin also proposed a mechanism for evolutionary change, his theory of sexual selection, and ascribes this force as the origin for many of the records of differences in ‘secondary sexual characters’. Sexual selection theory is fundamentally grounded in the idea that reproductive success varies amongst individuals within a population, and this drives the evolution of traits that maximize reproductive success over the course of an individual’s lifetime [11]. Classic examples of this come from several species of large mammals, such as the elephant seal, where male reproductive success is highly skewed; a few individuals siring the majority of the offspring in any 1 year. The skewed distribution in male reproductive success depends to a large extent on traits such as size of ornaments or weapons or overall body size [11].

**Figure 1:**
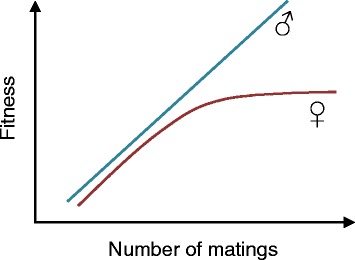
**Bateman gradients.** For males, fitness (in terms of reproductive output) is a simple linear function of the number of matings (or investment made in reproduction). For females however, the function is one of diminishing returns as fitness reaches a limit, at least over the short term, at an intermediate number of matings.

Sexual selection is a likely driving force for many examples of sexual dimorphism, particularly the weaponry and display traits that Darwin was interested in. But for other traits not used in male-male competition or mate choice, the explanation is less obvious. The mechanisms by which traits become sexually dimorphic, whether via sexual or natural selection, are still not fully resolved, although evolutionary models have been proposed based on sex-specific differences in selection and changes to the genetic architecture [12]. Essentially, differences in selection experienced by the two sexes are rooted in anisogamy (unequal gamete size). This as most fundamental of all sexual dimorphisms sets the stage for unequal investment by the two sexes in reproduction. Female reproductive success is limited by resource availability and acquisition, whereas male reproductive success is limited by access to mates and the number of fertilizations and may therefore be more variable. One way of visualizing this is to plot so-called Bateman gradients for the two sexes (Figure 1; named after the biologist A. J. Bateman who first studied them [13])—these show how fitness changes for each sex in terms of reproductive output as a function of number of matings.

Males and females maximize their lifetime reproductive success by employing different life-history strategies, and as a result, natural selection can act on shared traits in sex-specific ways. In some cases, the selection acting on shared traits may be so divergent that they are in opposite directions, this form of selection is termed sexually antagonistic selection [14]. For the genetic loci underlying any given shared trait, there may be intralocus sexual conflict (IASC) over which alternative alleles are favoured by selection in the two sexes [15]. At a genome-wide scale, there is now clear evidence that IASC exists in a variety of taxa [16]. It should be noted that although selection may operate in opposite directions in the two sexes, IASC is only realized when the intersexual genetic correlation for the trait (r_MF_) is in the opposite direction to selection [17]. Furthermore, the strength of the genetic correlation between the sexes can be taken as an indication of how readily sexual dimorphism will evolve, given diverging or opposing selection [12], (see below).

**Figure 2:**
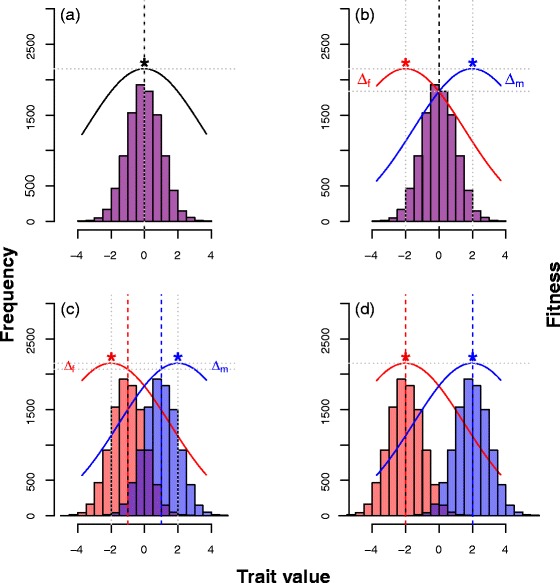
**Evolution of sexual dimorphism over four stages.** Each panel shows the frequency distribution of trait values for a hypothetical population (females (red), males (blue), overlap (purple)) and fitness surfaces (solid lines). Mean phenotypic trait values given by dashed vertical lines, optimum trait values given by asterisks, where fitness is maximized. **(a)** The trait experiences stabilizing selection to a single optimum trait value and the trait is sexual monomorphic; **(b)** the trait experiences sex-specific selection (red and blue fitness surfaces and optima) but is sexually monomorphic. As a consequence the population experiences a gender load (sum of Δ_f_ and Δ_m_), which is the difference between the maximum possible fitness (upper horizontal gray dotted line) and the fitness achieved by the population mean (lower horizontal gray dotted line); **(c)** the trait experiences sex-specific selection but has evolved sexual dimorphism, the population therefore experiences a reduced gender-load; **(d)** the trait experiences sex-specific selection but since the extent of sexual dimorphism matches the fitness optima, the gender-load has been eliminated.

According to sex-specific or sexually antagonistic models of evolutionary change, the evolution of sexual dimorphism can be broken down into four conceptually distinct stages [18]; see Figure 2a–d. The first stage is when a shared trait is in its ancestral monomorphic state (Figure 2a). While it is possible that newly emerging traits or new mutations can have directly sex-limited phenotypes (e.g. Y-linked loci, or traits derived from or associated with those already sex-limited), for the majority of cases, it is likely that they are manifest in both sexes. As a result, in this first phase, it is also likely that the genetic correlation between the sexes for any given novel trait is positive.

A trait will remain in a sexually monomorphic state unless selection acting on it becomes sex-specific, which could be caused, for example, by a change in environment or pre-existing sex-specific effects. If selection on the trait does become sex-specific, then the trait enters the second stage, where constraints in the genetic architecture of the trait may prevent it from becoming sexually dimorphic (Figure 2b). This may be because the trait exhibits a perfect intersexual genetic correlation (r_MF_ = 1) or because the genes underlying the trait function in other contexts (i.e. pleiotropically) and thereby constrain evolutionary change [19-21]. The constraint to evolutionary change has important consequences for a population experiencing sexually antagonistic selection. In effect, the mean trait value of either sex is displaced from its optima. This population level shortfall in fitness has been termed the sexual dimorphism, or gender-load [22], which has been empirically demonstrated by experimental evolution studies, e.g. [23]. The gender-load will be at its greatest for traits in this second stage, i.e. when they are sexually monomorphic but experience sex-specific selection pressures [24], assuming the sex-specific optima do not later change.

The third stage occurs when the genetic architecture of the trait allows it to evolve some limited degree of sexual dimorphism, i.e. the gender-load is not diminished entirely (Figure 2c). The changes in trait architecture between the sexes allow a relaxation of the intersexual genetic correlation (r_MF_ <1). There are several proposed mechanisms by which the genetic architecture of a shared trait can become at least partially sex-specific. These include the evolution of sex-specific epigenetic imprinting ([25], e.g. sex-dependent methylation [26]), sex-linked modifiers (hormonal or genetic [14]), alternative splicing of transcripts [27], plus more major genetic modifications such as gene duplication [28,29] or translocation to sex-chromosomes [30]. There is some evidence in support of some [16,19,31] but not all [32], and it is not clear which ones predominate or whether there are other mechanisms. More complex possibilities, such as sex-specific imprinting that influences the dynamics of epistatic loci [33], have not been investigated empirically.

The final stage occurs when the mean phenotypic value of the trait in both sexes is aligned with the sex-specific optimal trait values (Figure 2d). At this stage, the gender-load is eliminated and no further trait evolution is predicted, since although selection is sex-specific, it acts in a stabilizing manner in both sexes. Full resolution of sexual antagonism may not be possible for a variety of reasons, and it is not known how common full resolution is. For example, evidence from *Drosophila melanogaster* indicates that while the vast majority of gene transcripts are sex-biased, only a minority of these show sexually antagonistic patterns of expression [34], indicating conflict resolution may be widespread at least in adult stages.

The evolutionary transitions between these four stages will not necessarily always be one way, from early to later stages, as reversals seem plausible. For example, if selection pressures on the focal or genetically correlated traits change due to environmental parameters changing. Furthermore, the rate at which traits evolve to become sexually dimorphic, thereby resolving the conflict, is not known [35], although some of the mechanisms for conflict resolution require extensive revisions to the genetic architecture and may take considerable periods of time to occur (e.g. gene duplications, translocations). Finally, such major changes to the genetics may mean that a trait does not experience all four stages, since it is likely that the form and strength of selection that a trait experiences may also change radically following gene duplication or translocation to a sex chromosome.

It should also be noted that this evolutionary model is univariate—taking a single trait and predicting its evolutionary trajectory based on its genetic architecture and how selection acts upon it. In reality, traits do not occur in isolation but form part of a multivariate space of quantitative traits. As a consequence, the evolutionary trajectories that can be predicted are likely to be modified due to constraints imposed upon them by a considerably more complex genetic architecture [21].

#### Sex-specific genetics and disease

Darwin discriminated between the effects of sexual and natural selection, suggesting that the evolution of sexual dimorphism via sexual selection was maladaptive [10], in the sense that it appeared to favour the evolution of unwieldy or costly traits that could not have evolved via natural selection. Darwin’s framework therefore implicitly includes the concept of a gender-load and the potential for sexually dimorphic adaptations to be harmful in some way to an individual’s fitness, in terms of reproductive output or survival. The link between loss of fitness and disease is not direct in this case and Darwin himself never made that link explicitly. It is also not clear whether he considered the possibility that a gender load (or equivalent concept) could arise due to sex-specific natural selection.

By definition, sexually antagonistic alleles experience purifying selection in one sex by reducing survival or reproductive output and may do so by contributing to an individual’s overall propensity to develop disease. More generally, current population genetic theory predicts that alleles with either sex-specific or sexually antagonistic effects on Darwinian fitness can achieve higher frequencies as well as account for a greater proportion of genetic variance than alleles with symmetrical deleterious effects [7]. Genetic variants with sex-specific effects could therefore be important determinants of disease predisposition and as a result, sex is likely to be an important factor to consider when exploring the underlying causative loci of disease [5,24].

In cases where selection is sex-limited (i.e. purifying selection only operates in one sex (see [23] for an experimentally enforced version) either because the trait is only expressed in one sex or because the pattern of inheritance is sex-limited), then the genome is expected to accumulate mutations with sex-specific effects. There is some evidence of this process occurring in the *D. melanogaster* mitochondrial genome [36], where thanks to its pattern of maternal inheritance, a sex-specific selective sieve is predicted to result in the accumulation of mutations that are deleterious to males only (so-called ‘Mother’s curse’ [37]). A recent study in humans has also found evidence for a similar effect at autosomal loci [38], where genes with highly male-specific (essentially sex-limited) patterns of expression showed twice as many deleterious alleles as those expressed in both sexes. This empirical evidence supports evolutionary models that predict higher population frequencies for alleles experiencing asymmetric selection pressures across the sexes [7]. These models also predict equilibrium frequencies for sexually antagonistic alleles to be even higher than for sex-limited alleles [7].

One way of detecting the signal of genes with sex-specific effects is to examine sex-specific trait heritabilities, that is defined within a quantitative genetic framework as the proportion of phenotypic variance within a sample of individuals of one sex that can be accounted for by differences between genotypes, i.e. genetic variation [39]. In the absence of any loci with sex-specific effects and assuming a common environment, heritability estimates should not significantly differ between the sexes. If they do, then it may indicate the existence of genes with sex-specific genetic effects and sex-specific genetic architecture. A recent review of estimates in humans indicates sex-specific heritabilities are common for a range of traits, although not a universal feature [24].

Heritability estimates however are only a snapshot of genetic and phenotypic variance present within a population at a single point in time. As such, they may not be a reliable indicator of sex-specific effects or may miss more subtle or complex genetic effects (such as maternal, dominance or epistatic effects in the case of narrow sense heritability, which is based only on additive genetic effects), and similar heritability estimates may be obtained even when the genetic architecture differs between the sexes. More convincing evidence would be if individual loci demonstrated sex-specific effects on phenotypes. In fact, recently, there has been a surge of discoveries from genome-wide association studies (GWAS) that have identified loci with sex-specific effects [24], (see below).

#### Sexually dimorphic human quantitative traits and diseases

Pathologies of sex-limited traits are obvious examples of sex differences in disease; being absent entirely in one sex. More generally, there is clear evidence for widespread sexual dimorphism for a range of common diseases, as well as several human morphological, behavioural and physiological parameters (as any issue of *Biology of Sex Differences* will testify). These include cardiovascular disease, asthma, autoimmune diseases, some neurological and psychiatric disorders, as well as some common birth defects and cancers [5].

As I outlined in the introduction, sex differences in disease may in part be attributable to underlying sex differences in circulating hormones or other sexually dimorphic traits. For example, body musculature in humans is a sexually dimorphic trait that arises due to sex differences in the levels of circulating hormones during puberty [40]. The dimorphism in this morphological trait has functional repercussions, where reduced muscle strength relative to body mass in women increases their risk of developing knee osteoarthritis, a pattern not seen in men [41].

There is nonetheless, evidence that some genetic factors contributing to human quantitative traits or disease risk act in sex-specific ways. A recent review of GWAS hits identified 33 autosomal loci having sex-specific effects on 22 traits including quantitative traits such as waist-height ratio and blood lipid levels, as well as Crohn’s disease and type II diabetes [24]. The majority of these loci were sex-limited in their effects, with a smaller number having sex-asymmetric effects. It is worth highlighting that the majority of these are quantitative traits not disease phenotypes. However, since the most powerful analytical approaches for investigating sex-specific genetic effects are only recently being implemented [24], the number of loci having sex-specific effects on disease is likely to expand. The influence of sex on disease penetrance has also been reviewed by Cooper et al*.* [42], which contains further examples.

However, detecting sex-specific loci is likely to be a methodological challenge, in part because it is in essence an interaction effect, which usually requires larger sample sizes to achieve sufficient statistical power [24,43]. The complex mixture of factors contributing to disease risk further complicates the endeavour. For example, like many common diseases, the risk of developing cardiovascular disease is dependent on a number of environmental and genetic factors. It shows sex-specific patterns of prevalence [5], which may be dependent on hormonal effects (male-biased until the menopause, then becoming female-biased), but risk is also dependent on environmental factors (such as diet and exercise) and other traits for which sex-specific genetic effects have been identified (e.g. low- and high-density lipoprotein; body fat composition). Obtaining a full picture of how sex-specific selection on all these factors combine into one overall risk score is therefore unlikely to be straightforward.

None of the loci identified so far exhibit sexually antagonistic effects, i.e. being a risk factor for one sex but protective for the other. An interesting case however comes from the *Drosophila* model, where there is some evidence that the tumor suppressor *p53* is a locus that experiences sexually antagonistic selection [44], since its expression reduces life span in females but extends life span in males, with the effects being dependent upon developmental stage and environmental factors [45].

#### Sexual antagonism in humans

Evidence of sex-specific or sexually antagonistic selection on shared traits in humans is also scant; in part this may be due to the difficulty in obtaining appropriate data. One example however is height, which for females in western societies is under negative selection [46], whereas for males selection is curvilinear, meaning that men of intermediate height achieve highest lifetime reproductive success ([47], see also [48]). Selection on height is therefore sexually antagonistic. Furthermore, Stulp et al*.* [49] were able to show that there is sexually antagonistic genetic variation for height within their study population. Human height is a sexually dimorphic trait and so the evidence that it also experiences sexually antagonistic selection raises the question of why height does not evolve to become more sexually dimorphic, thereby resolving the conflict and eliminating the gender-load? One possible explanation is that the large number of loci segregating for variation in height [50,51] makes it difficult for a sex-specific genetic architecture to evolve due to the complex genetic correlations between the individual loci.

An alternative explanation is that since height is genetically correlated with other traits that experience different selection pressures, these genetic correlations constrain the evolution of greater sexual dimorphism [48]. A multivariate analysis of height and other size-related traits indicated that there was a negative genetic correlation with height in females and total cholesterol in males [48]. This indirect negatively pleiotropic relationship could therefore act as a constraint on the evolution of both traits in the two sexes. The complex nature of the genetic architecture of many traits may therefore hinder the resolution of sexual antagonism [19].

A second line of evidence comes from a Finnish longitudinal study where although phenotypic selection over the timing and rate of reproduction were found to be divergent between the sexes, the genetic correlations for these traits with fitness were not [52]. These results therefore suggest that no further sexual dimorphism is expected to evolve for these traits in this population. The authors suggest that cultural norms within this traditional population, which enforce strict monogamous sexual relationships, have constrained the variability in reproductive success and therefore reduced the opportunity for genetic variation in reproductive success to be expressed. This would mean that the genetic correlations between the timing and rate of reproduction might have been more divergent in populations where monogamy is not culturally enforced.

Together these studies offer a rather patchy view of sex-specific selection in human populations. They also highlight the considerable difficulties in obtaining suitable data, the ideal being a multigenerational study with a known pedigree and paternities, without strong cultural constraints on reproductive success [52]. These kinds of data may well already exist or possibly could be collated in the future, with genomic technologies being applied at a population scale combined with records of reproductive success, but this will undoubtedly raise ethical dilemmas if, for example, paternity is to be assigned based on genetic data.

## Conclusions

Evolutionary theory can provide a conceptual framework within which sex differences in human physiological or disease phenotypes can be understood. Sex differences in themselves can act as risk factors for disease, but there is increasing evidence that genetics plays a role in contributing to quantitative traits and disease risk in contemporary human populations. Although there are no concrete examples at present of sexually antagonistic loci contributing to disease risk, they are predicted to occur at higher frequencies than alleles with sex-limited effects, for which there is already evidence. Furthermore, while evidence that sexually antagonistic selection operates in humans is rare, there are recent examples that have demonstrated both selection and genetic variation can be sexually antagonistic. Overall, sex-specific and sexually antagonistic selection is clearly relevant to our understanding of the origins of human phenotypes, including disease, and this understanding could provide particular benefits to shaping therapies to the individual.

## Abbreviations

GWAS: Genome-wide association study
IASC: Intralocus sexual conflict
